# A systematic review of the effects of residency training on patient outcomes

**DOI:** 10.1186/1741-7015-10-65

**Published:** 2012-06-28

**Authors:** Renée M van der Leeuw, Kiki MJMH Lombarts, Onyebuchi A Arah, Maas Jan Heineman

**Affiliations:** 1 Professional Performance Research Group, Department of Quality Management and Process Innovation, Academic Medical Center, University of Amsterdam, Amsterdam, the Netherlands; 2 Department of Epidemiology, UCLA Fielding School of Public Health, University of California Los Angeles (UCLA), Los Angeles, California, USA; 3 UCLA Center for Health Policy Research, Los Angeles, California, USA; 4 Department of Obstetrics and Gynecology, Academic Medical Center, University of Amsterdam, Amsterdam, the Netherlands

## Abstract

**Background:**

Residents are vital to the clinical workforce of today and tomorrow. Although in training to become specialists, they also provide much of the daily patient care. Residency training aims to prepare residents to provide a high quality of care. It is essential to assess the patient outcome aspects of residency training, to evaluate the effect or impact of global investments made in training programs. Therefore, we conducted a systematic review to evaluate the effects of relevant aspects of residency training on patient outcomes.

**Methods:**

The literature was searched from December 2004 to February 2011 using MEDLINE, Cochrane, Embase and the Education Resources Information Center databases with terms related to residency training and (post) graduate medical education and patient outcomes, including mortality, morbidity, complications, length of stay and patient satisfaction. Included studies evaluated the impact of residency training on patient outcomes.

**Results:**

Ninety-seven articles were included from 182 full-text articles of the initial 2,001 hits. All studies were of average or good quality and the majority had an observational study design. Ninety-six studies provided insight into the effect of 'the level of experience of residents' on patient outcomes during residency training. Within these studies, the start of the academic year was not without risk (five out of 19 studies), but individual progression of residents (seven studies) as well as progression through residency training (nine out of 10 studies) had a positive effect on patient outcomes. Compared with faculty, residents' care resulted mostly in similar patient outcomes when dedicated supervision and additional operation time were arranged for (34 out of 43 studies). After new, modified or improved training programs, patient outcomes remained unchanged or improved (16 out of 17 studies). Only one study focused on physicians' prior training site when assessing the quality of patient care. In this study, training programs were ranked by complication rates of their graduates, thus linking patient outcomes back to where physicians were trained.

**Conclusions:**

The majority of studies included in this systematic review drew attention to the fact that patient care appears safe and of equal quality when delivered by residents. A minority of results pointed to some negative patient outcomes from the involvement of residents. Adequate supervision, room for extra operation time, and evaluation of and attention to the individual competence of residents throughout residency training could positively serve patient outcomes. Limited evidence is available on the effect of residency training on later practice. Both qualitative and quantitative research designs are needed to clarify which aspects of residency training best prepare doctors to deliver high quality care.

## Background

It is globally understood that high quality and safe patient care can only be provided if doctors are well prepared for this task through (residency) training [1]. Worldwide, medical educationalists and clinicians involved in residency training are focused on the improvement of education through modernizations including implementing competency based learning and assessing and advancing the quality of residents' education through accreditation standards [2,3]. Ultimately, societies need to know if it matters how, where and by whom doctors were trained.

Quality of care improvement initiatives have focused on finding solutions to ensure quality and safety in health care services. Research reports underscore the effectiveness of quality improvement initiatives in bettering patient outcomes [4,5]. However, it is not yet known to what extent residency training influences patient outcomes [6]. This is surprising given that there is a shared belief that quality and performance initiatives encourage life-long learning, which starts during undergraduate medical education (UGME) and continues through residency training. Research in medical education conducted to evaluate the effects of changes to residency training typically focuses on educational outcomes and does not include patient outcomes [7,8]. Research in UGME has focused more successfully on the link between medical education and patient outcomes. For example, scores achieved by medical students on qualifying examinations before licensing can be linked to later complaints to medical regulatory authorities [9,10]. In addition, professional behavior in both medical school and residency training has been correlated with later disciplinary action by medical boards [11,12]. Given the indisputable link between training and care delivery in daily practice, we would expect to find a vast amount of research focusing on the link between residency training and patient outcomes, to investigate and explain the relationship between the various aspects of training and their impact on patient care.

In daily practice, a resident is a 'learner' while being responsible for patients as a 'provider of care'. Thus, it is pertinent to know whether care delivered by residents is of at least equal quality to that delivered by faculty, or if it introduces a risk for patient care. Once training is completed, residents are expected to be well prepared to deliver a high quality of care. It is, therefore, essential to assess aspects of residency training through patient outcomes to evaluate the direct and future effects of global investments made in training programs. We assume that patient care provided during and after residency training will benefit from residents being well-trained. To our knowledge, outcomes of patient care delivered by residents during and after residency training have not been comprehensively studied in a systematic review. For that reason, we systematically reviewed recent literature on the broad research question: 'What is the effect of (aspects of) residency training on patient outcomes?'.

## Methods

### Data sources and searches

The primary data sources for this review were electronic databases MEDLINE, Cochrane, Embase and the Education Resource Information Center (ERIC). Databases were searched from December 2004 until February 2011 to place our review in the context of recent modernization efforts in residency training, work-hour restrictions and implementation of competency based learning in residency training.

A preliminary search was conducted with the assistance of a senior librarian to specify our keywords and optimize the search strategy. Databases were searched using keywords for both free text and Medical Subject Heading (MeSH) terms on the subjects of residency training and patient outcomes. In addition to the electronic search, reference lists from selected articles were later searched manually to obtain any additional relevant studies. We defined residency training as the training of residents, specialist registrars or trainees to become a specialist, consultant, general practitioner, family physician or faculty. The following keywords described residency training in our search string: Education, Medical; Teaching; Training, Clinical; Residency/Resident; Internship; Consultant; Faculty, Medical; Alumni. We added general and commonly used patient care outcome measurements to our search using the keywords: Outcome, Assessment, Clinical; Quality of Care; Safety; Complications, Postoperative; Surgical Wound Infections; Patient Readmission; Reoperation; Length of Stay; Iatrogenic Disease; Mortality, Hospital; Adverse Events; Patient Satisfaction. No language restrictions were applied. The search was limited to exclude comments, editorials or letters. The complete search string can be found in Additional file 1.

### Study selection

Clearly irrelevant articles were excluded based on the title and abstract by one reviewer (RML). Two independent reviewers (RML, KMJMHL) then assessed the title and abstract of all remaining articles for relevance to the study. If abstracts were unavailable, full-text articles were retrieved to assess relevance. After this selection of articles, all full-text articles were retrieved to examine compliance with the inclusion criteria. Any disagreement in the assessment of articles was resolved through discussion within the review team.

Studies were included if they explicitly related residency training to patient outcomes of residents' care. We included studies with an educational intervention or comparison and clearly defined and reported patient outcomes. Residents and faculty of all levels of experience were included if they were either participant or comparator. All types of study designs were included. Studies were excluded if the intervention was not educational in content (for example, duty hour reform) or if outcome measures were not directly related to patients (for example, knowledge or skill tests or performance indicators). Other reasons for exclusion were other types of education (such as dental, undergraduate or continuing medical education), non-research designs (for example, commentary) or participants who were not targeted as subjects of this systematic review (for example, medical students or nursing home residents).

### Data extraction and quality assessment

The review team agreed upon a data extraction form, which enabled one reviewer (RML) to extract data from included studies. Data extraction was regularly discussed and checked with other members of the review team. Information about participant characteristics, applied teaching interventions, patient outcomes, additional outcome measures, effect of residency training on patient outcomes, and study design were extracted. Review Manager 5 was used to collect data [13]. The quality assessment of included studies was performed using the validated Medical Education Research Study Quality Instrument (MERSQI) [14,15]. Two independent reviewers assessed the study quality of 10 articles to reach consensus; thereafter, one reviewer (RML) could complete the MERSQI for the remaining studies. The MERSQI enabled us to assess and compare the quality of all included studies by calculating the final MERSQI score as the sum of all scores, corrected for 'not applicable' items such as the internal structure, content and relation to other variables of measurement instruments.

### Data synthesis and analysis

If the data are suitable, we will perform a meta-analysis to synthesize and pool research findings using effect measures of studies with related research hypotheses. However, if the results are too heterogeneous, we will describe all study outcomes using a narrative analysis and a construction of subgroups based on primary objectives of studies to clarify study results and draw conclusions.

## Results

**Figure 1:**
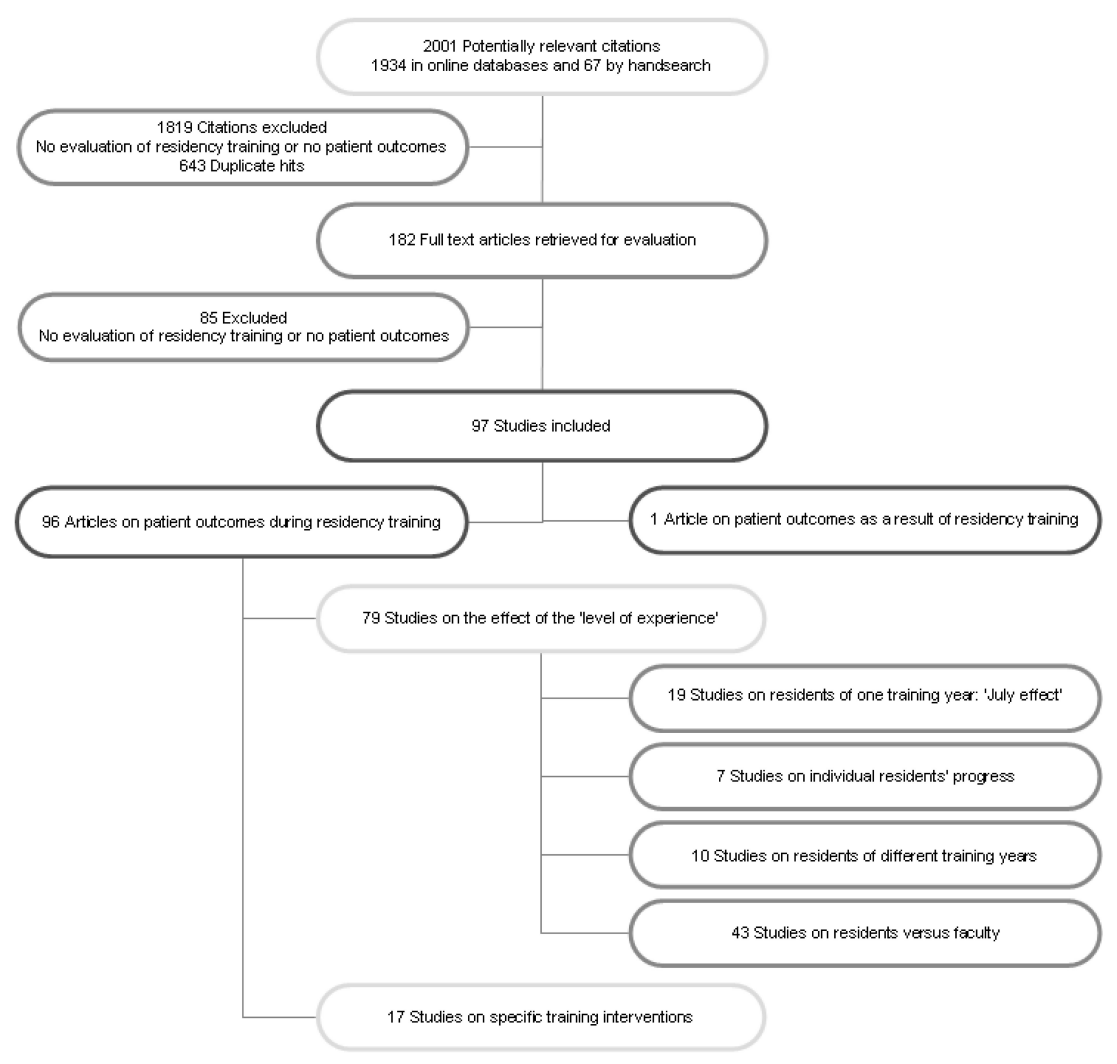
**Disposition of the articles found in the literature on the link between residency training and patient outcomes**.

We identified 2,001 citations, 1,934 citations by the literature search and 67 additional citations from a manual search. The broad selection of articles by title and abstract led to the retrieval of 182 potentially eligible studies. After a full-text review of the 182 studies, nine studies had to be discussed by the review team to reach consensus on inclusion or exclusion. A total of 97 studies met the inclusion criteria of this review. All studies were published in English. The selection process and subsequent categorization is summarized in Figure 1.

### Study design and quality

Because we included all types of study design, including a variety of different interventions and participants from different disciplines at all levels of training, the degree of heterogeneity was too great for any quantitative analysis of the data. The formation of subgroups also did not allow us to perform a meta-analysis. Therefore, we descriptively report study outcomes, with detailed quantitative information on individual studies available in the tables in Additional file 2. Study quality ranged from 9 to 15.6 (mean 12.9) on the MERSQI scale ranging from 4.5 to 18.

### Categorization of studies

Ninety-six studies evaluated the relation between resident participation in patient care and patient outcomes during residency training (Tables S1a to S1d and S2, Additional file 2) and one study focused on patient outcomes post-residency training, when residents practiced as independent faculty (Table S3, Additional file 2).

In the largest group of studies during residency training, two categories could be defined: describing patient outcomes as a result of either the level of experience of residents (79 studies; Tables S1a to S1d, Additional file 2) or specific training interventions (17 studies; Table S2, Additional file 2). To clarify the effects of the level of experience of residents on patient outcomes, the 79 studies were further divided into four subcategories according to the seniority of residents. First, 19 studies compared new residents commencing their training at the start of the academic year (July/August) to other months of the year (Table S1a, Additional file 2). Second, seven studies evaluated the effect of individual progress of residents on patient outcomes (Table S1b, Additional file 2). Third, 10 studies clarified the progression of residents through residency training by comparing patient outcomes of residents of different training years (Table S1c, Additional file 2). Fourth, 43 studies used patient outcomes of those cared for by faculty as a 'gold standard' to evaluate patient outcomes of residents' care (Table S1d, Additional file 2).

### Nineteen studies comparing the start of the academic year to another time of year (Table S1a, Additional file 2)

The first subcategory includes 19 studies investigating the impact of inexperienced residents on patient outcomes at the start of the academic year, also referred to as the July effect [16-34]. Of the 19 studies evaluating a July effect, 14 mostly surgical studies reported no differences in patient outcomes compared with other months of the year (risk-adjusted) [16-20,22-26,31-34]. Of the five remaining studies, one study reported no difference in mortality in July, but potentially preventable complications did occur more often in July, although numbers were too small to allow for effective root cause analysis [27]. Another study reported a higher risk-adjusted mortality in July, which progressively decreased over the course of the year [21]. In a study on medication errors, the July effect could partially explain a spike of fatal medication errors [29]. One study reported a small July effect on outcomes related to cerebral shunt surgery in children and emphasized the need for supervision [28]. Another study showed a reduction in resident scores on a patient satisfaction questionnaire around July [30].

### Seven studies evaluating the individual progression of residents using patient outcomes (Table S1b, Additional file 2)

In the second subcategory on the level of experience of residents, the individual progress or 'learning curve' of residents was covered by seven studies [35-41]. These studies refer to the progress individual residents make during their training or to the number of interventions they need to perform to reach a benchmark performance for specific procedures. Studies aimed to discover a plateau in the residents' learning curve in selected cases [40,41], under supervision [35,37], or with a stepwise approach [36,38,39]. Of the seven studies, six surgical studies (general surgery and obstetrics and gynecology) describe a decrease in operation time between the first and last cases performed by residents [35-38,40,41]. One emergency medicine study reported improved residents' performance on bedside ultrasonography [39].

### Ten studies comparing residents of different training years (Table S1c, Additional file 2)

Ten studies in the third subcategory evaluated patient outcomes between residents of different training years [42-51]. Six studies showed that more senior residents had better patient outcomes or higher patient satisfaction scores compared with junior residents [42-44,46,47,50]. One study showed that morbidity and mortality were not adversely affected by residents working under different levels of supervision [45]. Airway management by residents was reported safe in a large multicenter study with adequate rescue options [51]. One study reported a non-significant trend between the seniority of the resident and improved patient outcome [48]. Although one prospective cohort study reported that more senior residents were involved in complications, this was likely secondary to their disproportionate roles in more difficult operations [49].

### Forty-three studies using faculty as a gold standard to evaluate patient outcomes of residents' care (Table S1d, Additional file 2)

The fourth subcategory of studies related to the level of experience of residents includes 43 studies comparing faculty to residents. Thirty-eight of these studies focused on surgical disciplines (aesthetic surgery, general surgery, thoracic surgery, orthopedics, urology, ophthalmology and obstetrics and gynecology) [52-89]. The remaining five studies were conducted in radiology [90,91], family medicine [92,93] and anesthesiology [94]. Thirty-one studies reported no statistically significant differences between faculty and residents on a wide variety of outcomes including mortality, morbidity and length of stay [52,54,56-58,61,63,67-69,73-90,92-94]. Although patient outcomes were similar, 12 of these 31 studies did report a significantly longer operation time for residents [61,67,69,74,77,78,81,83,84,87-89]. Nine studies reported negative outcomes of residents' involvement in patient care [53,55,59,60,62,65,66,71,91]. Of these nine studies, five studies found significant differences between faculty and residents [53,55,59,60,91]. Residents' cases resulted in a modestly elevated blood loss during surgery without clinical consequences [53], radiographic or clinical leaks after esophagectomies without a higher take-back rate [55], a small but significantly higher take-back rate after cardiac operations [59], a discrepancy rate of 13.6% for residents' preliminary interpretations of radiology reports [91], and higher morbidity rates and length of stay for patients cared for by residents working without supervision [60]. Of the other four studies, two compared their outcomes to results in the literature [62,65], one did not formally train their residents [71] and another study reported the negative impact of residents' assistance in laparoscopic gastric bypass surgery compared with fellow or attending level assistance [66]. Three studies reported negative outcomes for faculty cases, likely caused by selection bias [64,70,72].

Sixteen studies highlight the need for supervision by showing similar patient outcomes for supervised residents [52,54,56-58,68,73,75,76,79,80,85,86,90,94] or worse patient outcomes for unsupervised residents [60,84]. Many studies compared faculty with residents in small settings with small sample sizes. However, two studies reported patient sample sizes > 5,000 patients with similar patient outcomes for both faculty and supervised residents after adjusting for case-mix [56,85].

### Seventeen studies evaluating the effect of specific training interventions (Table S2, Additional file 2)

The second category, of specific training interventions during residency training, comprised 17 studies that evaluated training programs on department level [95-111]. These specific training interventions were investigated in six medical [97,102,107-109,111], five surgical [98-100,103,105] and three intensive care [95,96,104] studies, and one each in psychiatry [110], anesthesiology [106] and all specialties [101]. Three randomized controlled trials were included in this category [101,107,111]. Two studies reported on audit and feedback intervention for residents providing diabetic care, which at first showed no differences in a small study [107], but improved diabetes mellitus control was found in a larger study [111]. Another randomized controlled trial in this category investigated a 40-hour role-play and feedback skills training program over eight months for residents, resulting in significantly higher patient satisfaction scores in the intervention group [101]. Seven of the 14 observational studies reported improved patient outcomes after implementation of a new training program [96,108], supervision [104,106] or simulator training and debriefing [95,97,105]. Six studies reported no difference in patient outcome [98-100,102,103,109] although one study did conclude that adherence to guidelines was better [109] and another study found that a redesign initiative reduced trainee workload and increased time for educational activities [102]. One study reported a drop in patient satisfaction after implementation of a training program in a psychiatric facility [110]. In conclusion, the studies in the category of specific training interventions showed improved or unchanged patient outcomes in the majority of studies and a drop in patient satisfaction in one study.

### One study describing post-resident patient outcomes according to where the resident trained (Table S3, Additional file 2)

One retrospective cohort study evaluated patient outcomes in relation to where the practicing physician completed their residency training [112]. In the only study on this subject, 43% of accredited obstetrics and gynecology residency programs in the USA (4,124 physicians from 107 residency programs) were evaluated. Training programs could be ranked by the maternal complication rates occurring in the patients of their graduates.

## Discussion

### Main findings

Residents need to provide high quality and safe patient care both during and after their residency training. Based on our review that explicitly focused on the effects of residency training on patient outcomes, we could not answer unequivocally whether residency programs can differentiate in producing 'better doctors'. Only one study related clinicians' training background to patient outcomes of the care they provide today [112]. The other 96 included studies provided more insight into the effects of residency training on patient outcomes. The start of the academic year is not without risk, but individual progression of residents as well as progression through residency training had positive effects on patient outcomes. Compared with faculty, care provided by residents resulted mostly in similar patient outcomes, when dedicated supervision and additional operation time are provided. Overall, specific training situations yield equal or improved patient outcomes, with additional educational benefits, compared with the original training situation.

### Limitations

Different sources of bias inherent to systematic reviews should be addressed. First, although we did not exclude non-English publications, all included studies were published in English, thus allowing for possible language bias. Publication bias is likely but difficult to assess in this heterogeneous body of evidence. Selection bias could be a possible limitation of our study design. However, two independent reviewers selected articles for inclusion by assessing the title and abstract and a limited selection bias in our approach is underlined by the fact that only nine articles had to be discussed after full-text retrieval.

Second, the studies included in our systematic review were too heterogeneous to perform a meta-analysis. To provide the reader with additional information alongside the narrative review of our results, the tables contain quantitative information on each individual study.

Third, we classified articles into categories based on their primary objective as described in the method section. We believe that the results are more easily read and understood with this categorization. Although we systematically assessed articles before assigning them into categories, categorization is always subject to discussion. However, only seven articles additionally reported on subjects of a category other than the one they were placed in [42,50,59,71,73,91,100]. Nonetheless, the results of these studies were consistent with the conclusions we drew for the categories they were not placed in.

### Explanation of results

Considering the recent modernization efforts in medical education and public attention to patient safety, we expected to find a shift towards research using patient outcomes in medical education. Although in daily practice residency training is inextricably bound up with patient care, current literature fails to relate the two explicitly. A study reported in 2001 demonstrated that in leading medical education journals only 0.7% of articles used patient outcomes [113] as a measure of performance. The anticipated change in medical education literature could not be proven given the fact that none of the articles in our review were published in solely medical education journals. This demonstrates a lack of the use of patient outcomes in residency training research. The fact that all but two studies were published in clinical journals shows that the clinical community has an interest in medical education. In most studies we reviewed, patient safety was seen as an important motivator of research into patient outcomes of residents' participation in patient care.

Given that new doctors have to learn, the practical significance of the differences observed and their acceptability to patients and those involved in residency training need explanation. From the results in this review, it is clear that the start of the academic year and residents' (individual) progression through residency training have the most potential as targets to improve patient outcomes of residents' delivered care. The start of the academic year has previously been reported to be a time for extra vigilance [114]. Although no firm conclusions about the degree of risk posed to patients can be made based on existing literature, Young *et al. *did conclude that mortality increases and efficiency decreases in hospitals during academic changeover [114]. Furthermore, individual progression through residency training requires the residents' level of experience (or inexperience) to be supported with adequate faculty supervision [36,51]. In studies comparing patient outcomes of residents' delivered care with patient outcomes from care provided by faculty, supervision was emphasized as an important part of residency training [84]. Furthermore, enhanced clinical supervision was found to be associated with improved patient- and education-related outcomes in a recent systematic review by Farnan *et al. *[115]. The intensity of faculty supervision depends also on the level of competency of residents, but determining residents' competency is a complex and multifactor process [116]. Operative exposure is essential for competency development as a certain number of operations need to be performed to reach benchmark standards [35-41]. The correlation between the residents' seniority and improved patient outcomes provides evidence for the positive effect of residency training on patient outcomes [42-48,50,51]. Finally, the overall positive patient outcomes of residents' care during residency training show that, within the complex situation of residency training, patients can be safely cared for by residents who are well supervised and given the time to learn. This could be reassuring to patients who might oppose being treated by residents.

### Implications for practice and research

As the above presented results show, there is a need for adequate supervision, patient selection, operative exposure, competency assessment and additional operation time to optimize residency training and ensure good patient outcomes. Faculty are primarily responsible for residents' learning during residency training and their support is essential, besides the additional time required. Therefore, it would be interesting to determine how much dedicated supervision and additional time is needed to ensure future health care workforce. The balance between the investment in teaching (time and money) and delivering care is something that is especially relevant for teaching hospitals. Although exploring differences between teaching hospitals and non-teaching hospitals was not the focus of this review, nine retrieved but excluded articles did compare them [117-125]. Overall, the teaching hospitals in these studies appeared to show better patient outcomes compared with non-teaching hospitals, predominantly on complex surgical procedures.

Clearly, the relationship between residency training and patient outcomes requires thorough investigation by both health care services and medical education researchers. In particular, studies evaluating the effect on patient outcomes after finishing residency training are currently lacking, since there was only one such study in this review [112]. Research on the training background of practicing physicians or prospective longitudinal follow-up of residents after finishing their residencies should be conducted. Although difficult, investigating causal factors that explain the relationship between residency training and patient outcomes can help us move forward in developing residency training. Furthermore, the effects of organizational aspects of residency training, like the impact of the teaching quality of the faculty on patient outcomes are lacking. Multicenter longitudinal databases of large student and resident cohorts exist, but they lack patient outcomes for individual doctors [126]. Cook *et al. *comprehensively describe longitudinal research databases facilitating the study of educational outcomes, taking patient outcomes into account. Intensifying collaboration between researchers and clinicians and encouraging the diversification of research perspectives should enrich clinical, health services and medical education research fields [127]. Therefore, both qualitative and quantitative research designs are needed to clarify which aspects of residency training best prepare doctors to deliver a high quality of care.

## Conclusions

The majority of studies included in this systematic review drew attention to the fact that patient care appears safe and of equal quality when delivered by residents. A minority of results pointed to some negative patient outcomes from the involvement of residents. We, therefore, conclude that adequate supervision, room for extra operation time, and evaluation of and attention to the individual competence of residents throughout residency training could positively serve patient outcomes. What is currently lacking is knowledge on how, where and by whom doctors should be trained to deliver high quality care in their careers after residency training.

## Competing interests

The authors declare that they have no competing interests.

## Authors' contributions

RML and KMJMHL designed the study and had full access to all of the data in the study and take responsibility for the integrity of the data, the accuracy of the data analysis, and writing the manuscript. OAA and MJH designed the study, discussed the results and critically revised the manuscript. All authors read and approved the final version of the manuscript.

## Pre-publication history

The pre-publication history for this paper can be accessed here:

http://www.biomedcentral.com/1741-7015/10/65/prepub

## Supplementary Material

Additional file 1 **Complete search string for seaching databases for relevant studies**. Click here for file

Additional file 2 **All tables (S1a to S1d, S2 and S3) containing detailed information on all individual studies**. Click here for file
