# Supplementary material for: A systematic review of the effects of residency training on patient outcomes
Source: BMC Med. 2012 Jun 28;10:65. doi: 10.1186/1741-7015-10-65 (PMC3391170; doi:10.1186/1741-7015-10-65)
Supplement: Additional file 1 — Complete search string for seaching databases for relevant studies. [file 1741-7015-10-65-S1.PDF]

# Additional file 1. Search string

("Education, Medical"[Mesh:NoExp] OR "education"[Subheading] OR medical education[tiab] OR teaching[tiab] OR clinical training[tiab]) AND ("Outcome Assessment (Health Care)"[MeSH:NoExp] OR "patient satisfaction"[MeSH Terms] OR "quality of health care"[Mesh:NoExp] OR "patient care"[Mesh:NoExp] OR "Safety/standards"[Mesh:NoExp] OR patient safety[tiab] OR patient outcome*[tiab] OR clinical outcome*[tiab] OR "complications "[Subheading] OR "Postoperative Complications"[Mesh] OR "Surgical Wound Infection"[Mesh] OR "Patient Readmission"[Mesh] OR "Reoperation"[Mesh] OR "Length of Stay"[Mesh] OR "Iatrogenic Disease"[Mesh] OR "Hospital Mortality"[Mesh] OR mortality[tiab] OR adverse events[tiab]) AND ((“internship and residency"[MeSH Terms] OR internship[tiab] OR residency[tiab] OR trainee*[tiab] OR residents[tiab] OR house officer[tiab] OR consultant[tiab]) OR ("faculty, medical"[MeSH Terms] OR medical faculty[tiab] OR alumni[tiab])) AND ("last 5 years"[PDat]) NOT ("Editorial "[Publication Type] OR "Letter "[Publication Type] OR "Comment "[Publication Type])
