# Supplementary material for: A systematic review of the effects of residency training on patient outcomes
Source: BMC Med. 2012 Jun 28;10:65. doi: 10.1186/1741-7015-10-65 (PMC3391170; doi:10.1186/1741-7015-10-65)
Supplement: Additional file 2 — All tables (S1a to S1d, S2 and S3) containing detailed information on all individual studies. [file 1741-7015-10-65-S2.PDF]

Table S1a. The effect of level of experience of residents on patient outcomes: Inexperienced residents at the start of the academic year: the July effect

First author and publication year	Setting	Specialty	Participants	Sample size	Teaching interventions	Comparison	Patient outcomes	Other outcome measurements	Effect	Study design	MERSQI score
Alshekhlee 2009 [16]	1045 hospitals, 2000 - 2005	Neurology	a 20% representative sample of all hospitals in the United States	377266 acute ischemic stroke patients were admitted	43% of patients were admitted to teaching hospitals	monthly trends were analysed	in-hospital mortality and thrombolysis	admission before or after 2003 (work-hour)	trend analyses showed no significant change in mortality for months of the year (*p*-value 0.25-0.93)	retrospective pre-post study	14.4
Anderson 2010 [17]	Hospitals included in the Nationwide Inpatient Sample, 1998-2003	Orthopedics	a 20% representative sample of all hospitals in the United States	324988 elderly patients hospitalized for intertrochanteric or a femoral neck fracture	July/August in were compared to other 2-months periods in teaching hospitals	2-months periods in nonteaching hospitals	in-hospital mortality, intraoperative and perioperative complication rates, length of stay	hospital charges and month of admission; potential confounders were age, sex and comorbidity status	in-hospital mortality rates were lowest or second lowest in July/August (below the mean mortality rate of 3.64%)	retrospective pre-post study	14.4
Ayas 2007 [18]	Two tertiary care teaching hospitals, 1999 - 2005	Intensive care	intensive care residents participate in 1 or 2 months rotations, 78% started on the first of every month	3548 patients admitted, 5816 central venous catheters (CVC) inserted	junior residents are supervised by senior residents and during daylight hours attendings and fellows were present	July and August were compared to other months of the year	pneumothorax (PTX) as an 'ICU-acquired diagnosis' or as a 'complication' occurring within 48 hours after a CVC was inserted in either the subclavian or the jugular vein	PTX rates at the first week compared to other weeks of the month	rates of PTX after catheter placement were not greater in July/August compared to other months (AOR 1.24, 95% CI 0.79 to 1.97, p=0.35)	retrospective pre-post study	12.6
Bakaeen 2009 [19]	44 Veterans Affairs cardiac surgery centers, October 1997 - October 2007	Thoracic surgery	medical/surgical trainees involved in the care of 70616 cardiac surgical patients	11975 cardiac surgical procedures between July 1 and August 31	residents rotated at various times throughout the year at different centers, July universally marked the beginning of the academic year	58641 patients underwent an operation between September 1 and June 30	operative time, length of stay, perioperative morbidity (including endocarditis, renal failure necessitating dialysis, mediastinitis, reoperation for bleeding, requiring a ventilator for longer than 48 hours, stroke, coma lasting longer than 24 hours, repeat cardiopulmonary bypass or mechanical circulatory support) and 30-day postoperative mortality	month of the year	early and later parts of the year were similar for both morbidity (AOR 1.01, 95% CI 0.96-1.07, *p*=0.67) and mortality (AOR 0.99, 95% CI 0.89-1.11, *p*=0.90)	retrospective pre-post study	14.4

Dhaliwal 2008 [20]	One Veterans Affairs medical center, October 1997 - April 2007	Thoracic surgery	one first year resident and one second-year (chief) resident working under appropriate supervision	242 cardiac surgical procedures between July 1 and August 31	throughout the academic year 2 cardiothoracic residents rotate in 4-months blocks starting on the first day of July	1431 patients underwent an operation between September 1 and June 30	operative time, length of stay, perioperative morbidity (including endocarditis, renal failure necessitating dialysis, mediastinitis, reoperation for bleeding, requiring a ventilator for longer than 48 hours, stroke, coma lasting longer than 24 hours, repeat cardiopulmonary bypass or mechanical circulatory support) and 30-day postoperative mortality	month of the year	the early period of the academic year was not significantly associated with morbidity (AOR 0.83, 95% CI 0.54-1.28, *p*=0.41) and mortality (AOR 0.28, 95% CI 0.07-1.19, *p*=0.09)	retrospective pre-post study	13.2
Englesbe 2007 [21]	14 academic and 4 community medical centers, 2001 - 2004	Surgical specialties	interns and residents starting in July	9941 major surgical procedures between July 1 and August 31	the 4 community hospitals contributed a relatively small number of cases because of their late enrolment	10313 patients underwent an operation between April 15 - June 15	30 day morbidity and mortality are endpoints used in the American College of Surgeons-National Surgical Quality Improvement Program (ACS-NSQIP)	month of the year	after controlling for potential confounding variables, there was a 18% higher morbidity risk and a 41% higher mortality risk for the early group versus the late group. A decreasing trends was found mortality (Regression coefficient 0.379, *p*=0.028)	retrospective pre-post study	14.4
Englesbe 2009 [22]	Hospitals with membership of the Council of Teaching Hospitals (COTH), 2003 - 2006	Surgical specialties	residents	26198 medicare patients between 65 and 99 years old undergoing 1 of 7 major surgical procedures in July	residents had significant part in overall patient care in COTH	294018 patients underwent 1 of 7 major surgeries in other months of the year	risk-adjusted 30-day mortality before discharge or within 30 days for seven procedures: coronary artery bypass grafting, carotid endarterectomy, abdominal aortic aneurysmectomy, esophagectomy, colorectomy, pancreatectomy, hip surgery	month of the year	no evidence for higher mortality rates in July for seven procedures with odds ratios ranging from 0.83 to 1.15 (95% CI 0.61-1.14 to 0.83-1.61)	retrospective pre-post study	14.4
Finkielman 2004 [23]	One academic tertiary medical center, October 1994 - September 2002	Intensive care	residents (mostly fresh graduates from medical school) starting in July	2728 patients admitted to one medical, two surgical or one multiple-specialty intensive care unit (ICU) in July	residents rotate in the critical care team for 4-5 weeks at a time under supervision of a second-year fellow	26356 patient were admitted to the ICU in other months of the year	Acute Physiology and Chronic Health Evaluation (APACHE) III score and predicted mortality, intensity of treatment, ICU length of stay, hospital mortality	month of the year, type of ICU	there were no statistically significant differences in adjusted mortality rates in July compared to other months odds ratios ranging from 0.94 to 1.33 (95% CI 0.73-1.20 to 1.05-1.69)	retrospective pre-post study	13.2

Ford 2007 [24]	217 mostly urban and large teaching hospitals, 1998 - 2002	Obstetrics and Gynecology	a 20% representative sample of all hospitals in the United States	26546 singleton deliveries and live birth admissions patients in July	hospitals were selected that were identified as by the NIS as 'teaching hospitals' and with publicly available data residents' participation on the labor floor was checked	272584 singleton deliveries and live birth admissions patients in July	postpartum hemorrhage, perineal and cesarean wound complication, anesthetic complications, third of fourth degree lacerations, bladder or urethral injury, cesarean section procedure, vacuum- and forceps-assisted vaginal deliveries, shoulder dystocia, infection of the amniotic cavity, rates of birth asphyxia and brachial plexus injuries	month of the year	no increase rate of operator dependent complications of delivery at teaching hospitals nationwide were seen in the month of July compared to the rest of the year unadjusted OR ranged from 0.91 to 1.08 (95% CI 0.78-1.05 to 0.75-1.52, *p*=0.178-0.841)	retrospective pre-post study	14.4
Garcia 2009 [25]	One university hospital, January 2000 - July 2004	Cardiology	two interns and two internal medicine residents under supervision of a board-certified internist	191 patients with acute coronary syndromes (ACS) and 93 patients with decompensated heart failure (HF) admitted between July 1 - September 30	residents actively participated in patient care and made discharge summaries and medication lists which were signed by the responsible attending physician. Residents rotated every 28 days	573 patients with ACS and 516 patients with HF between October 1 - June 30	length of stay, in-hospital mortality, for ACS patients who underwent percutaneous coronary interventions or bypass surgery myocardial infarction or stroke were also measured	month of the year and process outcomes at the time of discharge: for ACS patients: aspirin, beta-blockers and statin use; for HF patients: beta-blocker or angiotensine-converting enzyme inhibitor or angiotensin receptor blocker use	in-hospital complications, length of stay and mortality were no different among patients admitted during July-September versus October-June (OR 0.93, 95% CI 0.67-1.3, *p*= 0.70)	retrospective pre-post study	13.2
Highstead 2009 [26]	One large urban Level I trauma center, 1998 - 2007	Surgery	size and composition of the trauma team was unchanged for the duration of the study period with 24-hour in-house trauma attending coverage	3967 trauma patients admitted in July/August	medical and surgical training programs include trauma and critical care	3626 trauma patients were admitted in April/May	in-hospital mortality, complication rate (neurologic, pulmonary, cardiac, gastrointestinal, genitourinary, vascular, infectious, skin/wound, iatrogenic, other), length of stay, intensive care length of stay	month of the year	outcomes were similar between patients admitted at the beginning compared with the end of the academic year (*p*-values ranging from 0.07 to 0.96)	retrospective pre-post study	13.2
Inaba 2010 [27]	One academic Level I trauma center, December 2001 - December 2006	Surgery	interns, junior and senior residents starting their rotation on the trauma service in July with 24-hour in-house attending trauma surgeon coverage	4030 injured patients were admitted in July or August	the majority of interns' and residents' learning is directly supervised, they do work independently on many tasks	4121 injured patients were admitted in May or June	hospital and intensive care unit length of stay, complications (and its preventability as reach by consensus on weekly Mortality and Morbidity conferences), mortality	month of the year	risk-adjusted mortality did not differ between the start and end of the academic year (AOR 1.1 (95% CI 0.81-1.5, *p*=0.516); (potentially) preventable complications did occur more at the start of the academic year (AOR 1.9, 95% CI 1.1-3.2, *p*=0.013), although numbers were too small to allow effective analysis of root causes	retrospective pre-post study	13.2

Kestle 2006 [28]	Trial data from 10 centers October 1993 - October 1995 (1), 14 centers May 1996 - November 1999 (2) and 11 centers 1989 - 2001 (3) were combined	Neurosurgery	residents starting in July	138 (1 and 2 combined); 490 (3) children with hydrocephalus undergoing cerebrospinal fluid shunt surgery in July or August	the amount of teaching and supervision of residents during shunt surgery is a potentially significant variable in the analysis of a July effect, which was not measured specifically in any of the datasets	599 (1 and 2 combined); 2578 (3) children with hydrocephalus underwent cerebrospinal fluid shunt surgery between September 1 - June 30	shunt survival (median duration of shunt lifespan), shunt failure, shunt infection, neurological deficits, wound infection, wound dehiscence, technical errors, death	month of the year	Only the incidence of wound dehiscence differed between shunts inserted in July and August and the rest of the year (*p*-value = 0.05)	retrospective pre-post study	14.4
Philips 2010 [29]	United States death certificates, 1979 - 2006	All	proportion of teaching hospitals in each county	fatal medication errors (deaths in which medication errors are recorded as the primary cause of death inside medical institutions)	American Hospital Association (AHA) surveys were used to identify counties containing major teaching hospitals	persons dying before reaching medical institutions	ratio observed to expected deaths for inpatient medication errors by month	July effect for fatal medication errors by hospital setting, July effect for fatal medication errors and for comparison causes of death inside medical institutions, July effect by cause of death for teaching hospital counties and for all other counties	fatal medical errors spiked in July, only in counties containing teaching hospitals. These findings were evident only for medication errors and not for other causes of death or for deaths outside medical institutions. No trends were observed for the observed number of deaths/expected number of deaths from medication errors in July (b=0.0003; t=0.104; $p>0.05$)	retrospective pre-post study	14.4
Resnick 2008 [30]	One university hospital, April 2005 - June 2006	All	residents	4764 patients were admitted and discharged in the same month	Accreditation Council for Graduate Medical Education (ACGME) residency program	different months were compared to evaluate seasonality	patient satisfaction was measured with the widely used Press-Ganey patient satisfaction surveys questioning patients on overall rating of care and their satisfaction with different health care providers including house staff	month of the year and evaluation scores of residents by faculty	Residents received lowest scores (82.4) in the end (June) and (82.8) beginning of the academic year (July) which was significantly lower compared to the scores (84.4) during the rest of the year ($p<0.05$)	retrospective cross-sectional study	11.5
Schroeppel 2009 [31]	One academic Level I trauma center, July 1, 2001 - June 30, 2006	Surgery	residents are supervised by attending trauma surgeon taking in-hospital call and are actively involved in patient care at all times	12525 patients were admitted with a blunt trauma	Surgical residents are intimately involved in the care of trauma patients from initial evaluation through discharge during 1 month rotations	different months were compared to evaluate seasonality	mortality, Injury Severity Score, 24-hour transfusion requirement, ventilator-support days, intensive care unit (ICU) days and length of stay	month of the year	there was no monthly variation in mortality (AOR ranged from 0.62 to 1.29, 95% CI 0.36-1.08 to 0.75-2.23), ICU days ($p=0.225$), ventilator-support days ($p=0.574$) or minutes in the resuscitation room ($p=0.497$)	retrospective cross-sectional study	12

Smith 2006 [32]	1045 hospitals, 1998 - 2000	Pediatric neurosurgery	a 20% representative sample of all hospitals in the United States	3002 craniotomies for tumor resection and 14975 shunt placements or revisions	Hospital teaching status according to the American Hospital Association Annual Survey of Hospitals	different months were compared to evaluate seasonality	in-hospital mortality rates, discharge other than directly home, neurological complications or hematomas related to the procedure, transfusions of packed red blood cells, length of stay	month of the year	July and August were not associated with more frequent mortality or adverse patient outcomes compared to other months for craniotomy (AOR ranged from 0.43 to 1.03, 95% CI 0.14-1.32 to 0.71-1.51, *p*>0.14) or for shunt surgery (AOR ranged from 0.81 to 1.44, 95% CI 0.48-1.37 to 0.77-2.71, *p*>0.24)	retrospective pre-post study	14.4
Soltau 2008 [33]	One university hospital, January 1991 - June 2004	Intensive care (neonatal)	pediatric and neonatal house staff supervised by 1 neonatal fellow who have 24-hour presence and 2 board-certified neonatal faculty on call	11285 infants (3445 premature and 7840 mature infants)	4 pediatric interns (1st year residents), 3 pediatric 2nd and 3rd year residents are given considerable freedom and latitude in clinical decision-making and management	different months were compared and two halves of the year (January through June and July through December)	intraventricular hemorrhage grades 3-4/periventricular leukomalacia (IVH), necrotizing enterocolitis > Bell stage 2 (NEC), bronchopulmonary dysplasia (BPD)	month of the year	mortality rates for both cohorts did not differ between July and other months of the year (*p*=0.49). For premature infants no differences between July and others months were found for IVH (*p*=0.20), NEC (*p*=0.63) or BPD (*p*=0.98)	retrospective cross-sectional study	12.6
Yaghoubian 2010 [34]	Two public teaching hospitals, 1998 - 2007	Surgery	surgical residents	766 patient with appendicitis were operated in July or August	junior residents often manage patients with appendicitis early in their training and as primary surgeon	3559 patients with appendicitis were operated in all other months	wound infection rate, rate of postoperative abscess drainage, length of hospital stay	month of the year	appendicitis outcomes were similar (*p*>0.1) in July compared to other months of the year, wound infection rate was found to be higher in July compared to April/May (*p*=0.02)	retrospective pre-post study	13.8

AOR Adjusted Odds Ratio; OR Odds Ratio; CI Confidence Interval

Table S1b. The effect of level of experience of residents on patient outcomes: The individual progress of residents

First author and publication year	Setting	Specialty	Participants	Sample size	Teaching interventions	Comparison	Patient outcomes	Other outcome measurements	Effect	Study design	MERSQI score
Ascher-Walsh 2007 [35]	University hospital, July 2000 - June 2005	Obstetrics and Gynecology	25 fourth year residents	206 hysterectomies	All residents performed laparoscopic supracervical hysterectomies for 10 consecutive weeks with one supervisor	Comparison of their first 2 cases and last 2 cases	pre ; intra ; post -operative values (demographics, indication for surgery, body-mass index, parity ; uterine mass, blood loss, surgical complications, operating room time ; length of stay, change in hematocrit, postoperative complications)	Differences in the amount of practice between first and last two were not assessed	operative time decreased (*p*<0.001) between the first and last two patients. Other outcomes were not significant *p*>0.29)	retrospective case-control study	13.2
Fok 2006 [36]	Tertiary obstetric unit, when the study was performed is not reported.	Obstetrics and Gynecology	10 trainees	500 caesarean sections	trainees operated as first assistant, then as primary surgeon with the supervisor as first assistant and after that; operated independently with an intern assistant	500 cases divided into 10 equal groups to compare the first 50 to the last 50 cases	incision-to-delivery time, total operative time, blood loss, apgar score, umbilical blood pH, neonatal intensive care admission, post temp, wound infection, urinary tract infection, length of stay	-	operation time decreased in the first 15 cases (*p*<0.027), incision-to-delivery time decreased in the first five cases (*p*=0.02) and operative blood loss decreased in the first ten cases (*p*=0.053) compared to following cases	retrospective case series	13.8
Grotenhuis 2008 [37]	Two university teaching hospitals, July 1992 to July 2007	Surgery	15 trainees	186 cases of laparoscopic cardiomyotomy	assessment of 1. overall learning curve using chronologically arranged groups, 2. the same but on individual level, 3. comparison of trainee and consultant	5 consultants	duration of the operation, incidence of perforations, conversion, reoperation, follow-up with a structured questionnaire for patients at 3 months, 1 year and 2 years postoperative assessing the satisfaction and improvement of dysphagia	experience (chronologically assessed); consultant or trainee under supervision as first surgeon	duration of operation was longer and conversion to an open procedure was also higher in first 20 cases (*p*<0.01). When compared to consultants, the length of operation was longer for trainees (93 versus 73 minutes respectively, *p*<0.01)	case-control study	14.5
Ibrahim 2008 [38]	One hospital, May 2000 - May 2006	Surgery	3 trainees	1000 laparoscopic cholecystectomy's (LC)	Residents were gradually trained in LC, they had completed their 3-year course of basic surgery	5 consultants with 200-300 LC	length of stay, duration of the operation, conversion, bile duct injuries, other complications (wound infection, infra umbilical incision hernia, pneumonia, mortality)	3 trainees were assessed as trainees (group 2) versus after their training when they were accredited surgeons (group 3) versus consultants (group 1)	longer duration of operation in trainees (*p*<0.0001). Retrospectively, 67 cases of laparoscopic cholecystectomy were found to be needed to reach similar outcomes as experienced surgeons	retrospective case series	12.6

Jang 2010 [39]	One urban academic hospital, December 1, 2004 - December 31, 2007	Emergency medicine	23 of 57 eligible residents, 11 postgraduate year (PGY) 3 and 12 PGY 4 residents	393 patients had an emergency department bedside ultrasonography (EUS)	2-week elective rotation in EUS after an introductory course on EUS in PGY 2 and 10 prior experience of EUS exams for obstructive uropathy	CT scans read by board-certified radiologists blinded to the EUS results	EUS diagnosis and calculated sensitivity, specificity and true positive EUS (positive EUS diagnosis associated with a CT diagnosis) measured for every 10 additional EUS performed by residents	-	Residents may be able to accurately assess for obstructive uropathy after 30 examinations to achieve 95% sensitivity and 92% specificity	prospective cohort study	10.8
Jensen 2009 [40]	A district general hospital, January 2003 - December 2007	Surgery	higher surgical trainees operated 21 cases under supervision	113 day-case laparoscopic Nissen fundoplication (LNF)	trainees performing day-case LNF in eligible patients	3 consultants	length of stay, complications (wound bleeding, wound infection, regurgitation, dysphagia), length of procedure, symptoms on follow-up (48h postoperative a telephone call to check up on the patients)	grade of the operating surgeon	operative time reduced between the first and latest 20 cases (65 versus 48 minutes, *p*=0.037). Numbers were to small to assess the grade of the operating surgeon	case series	11.4
Lin 2010 [41]	National university hospital, January 2002 - December 2007	Surgery	6 unsupervised residents with at least 2-4 years of training who have been found proficient in independent laparoscopic appendectomy	306 laparoscopic appendectomies	independently performed acute laparoscopic appendectomies by residents (cases with assistance from a senior surgeon were excluded, n=26)	first 20 and last 20 cases	complications, operative duration, length of stay, conversion	first 20 and subsequent (last) 20 cases of each resident	operative duration (88 versus 71 minutes, *p*=0.005) and complication rates (8.3% versus 2.6%, *p*=0.04) decreased with increasing experience of the residents	retrospective case series	12.6

Table S1c. The effect of level of experience of residents on patient outcomes: Residents of different training years

First author and publication year	Setting	Specialty	Participants	Sample size	Teaching interventions	Comparison	Patient outcomes	Other outcome measurements	Effect	Study design	MERSQI score
Daetwiler 2007 [42]	One hospital, June 1999 - May 2005	Surgery	12 trainees of different levels of experience (A 5 lap and 10 open; B 5 lap and > 10 open; C 6-10 lap; D 11-30 lap; E >31 lap)	262 sigmoid colectomies	all residents gained considerable training experience in general laparoscopic sigmoid colectomy (tutorial course, >75 basic and assisting in >20 advanced laparoscopic procedures)	comparison between different levels of experience of residents and comparison to one senior surgeon	duration of the operation, blood loss, intra- and postoperative complications, conversion, length of stay, 30-day mortality, readmission rate	level of experience in laparoscopic colectomy, divided in 5 groups (level A-E) with increasing numbers of open/lap operations. From level C, residents were encouraged to perform the operation independently with an intern	operation time and blood loss decreased with increased level of experience ($p<0.01$), however, no association between level of experience and operative complications was found (AOR ranged from 0.28 to 1.89, 95% CI 0.04-1.80 to 0.59-6.05, $p>0.179$)	prospective case series	12.6
Filippi 2008 [43]	1 Dec 2006 - 31 May 2007	Radiology	radiology residents gave preliminary interpretations during after hours	361 magnetic resonance angiographic examinations	review of discrepancies between the preliminary reading (resident) and the final report (attending) by two experienced neuroradiologists, blinded to clinical data of the patient but not to the indication	attending neuroradiologists' final report	classified as false positive (normal as abnormal) ; false negative (failure to diagnose an abnormality) ; true-positive (infarct as an infarct) ; true-negative (normal as normal). Readmission, intensive care admission, new surgery, initiation of med treatment, new examinations were used as a change in clinical management	residency years 1-4 (n=16)	higher discrepancy rate for first year residents compared to other residency years ($p=0.04$) without clinical consequences as reported by direct care providers	retrospective case series	10.8
Goldmann 2008 [44]	University hospital, 1 July 2005 - 31 Dec 2006	Anesthesiology	first and second year residents	2114 cases of use of a laryngeal mask	use of the proseal laryngeal mask airway (PLMA) or classic laryngeal mask airway (CLMA)	attending anesthetist and third - fifth year residents	PLMA use related critical incidents, postoperative status of the patient, ASA and anesthesia classifications, duration of the operation, change to endotracheal tube, use of positive end-expiratory pressure, use of gastric tube, persistent airway leakage, adverse event (airway obstruction, hick-up, regurgitation, aspiration, hypoxemia, complaints of the patient broncho/laryngospasm and others)	resident year, attendings' identification number, attendings' experience with CLMA and PLMA.	1st and 2nd yr residents had a higher incidence of adverse events (15.2%) than 3-5yrs (10.1%) or attending anesthesiologists (9.5%) ($p<0.001$).	prospective case series	9

Itani 2005 [45]	120 VA hospitals, 99 of which with an academic affiliation. October 1998 - September 2004	Surgery	residents operating without an attending in the operating room, but immediately available	610 660 surgical cases	surgical residency program	Supervised residents or attending alone	mortality and morbidity at 30 days and specific postoperative complications	level of attending supervision recorded by the nurses in the operating rooms	attending being immediately available versus increased levels of supervision showed no difference in morbidity (AOR ranged from .66 to 1.01, 95% CI 0.595-0.720 to 0.863-1.164) or mortality (AOR ranged from 0.72 to 1.03, 95% CI 0.594-0.858 to 0.842-1.256)	retrospective case series	15
Krugman 2009 [46]	19 paediatric resident continuity practices, 1 week in May 2004 and 1 week in June 2004	Paediatrics	19 paediatric residency continuity clinics from Continuity Research Network (CORNET)	360 residents from year 1	a survey to assess differences between residents from year 1 and year 3 as perceived by parents of children who are cared for in continuity clinics	347 residents from year 3	parental perception of quality of care scores on a validated 23item list (the parental perception on primary care (P3C))	first year or third year paediatric resident	parents perceive residents yr 1 and 3 as providing high quality primary care; the duration of the relation accounted for a different mean score (yr1 77.2, 95% CI 75.0-77.2 and yr3 82.0, 95% CI 80.0-83.9, p=0.002)	case series	11.5
Lee 2008 [47]	General hospital January 1, 2002 - December 31, 2006	Intensive care	2 groups: junior (years 1-2) and senior residents (years 3-4)	2274 patient from a surgical (8bed) and neurological (8bed) intensive care unit (ICU)	surgery residents for 1 to 3 month at a time covering both ICU's under supervision (direct and at night time on the phone) of an attending	both groups were compared	days of ICU stay, mortality	training year of residents, admission day, supervising attending, specialty to which the patient was admitted, diagnosis, operative procedure	mortality was higher in patients cared for by junior residents (24.7% versus 17.7%, p=0.002), Due to operation schedule constant supervision for residents could not be guaranteed	retrospective cohort study	13.8
Lowe 2006 [48]	2 emergency departments (university and peripheral), May 2004 - August 2004	Emergency medicine	junior residents (year 2 and 3)	132 patient rated cosmesis of their scar after emergency department wound care	1 hour training (half hour theory, half hour practicing on pig trotters)	senior residents (>year 3) and anyone with more experience	patient and wound characteristics, wound management techniques, complications (infection, wound gaping, re-suturing) and visual analog scale cosmetic outcomes at 14d and 3 months	practitioner seniority	cosmetic score did not differ between junior and senior residents at 14d (p=0.15) and 3 months (p=0.30)	case series	13.5
Nieder 2005 [49]	3 teaching hospitals, November 2003 - October 2004	Urology	supervised residents (year 2-5) under specialists' supervision	173 transurethral bladder tumor resection (TURBT)	supervising faculty determined residents' or fellows' participation. Operations performed entirely by faculty were not included in the analysis	fellows (year 6-7) under specialists' supervision	intraoperative complications were recorded by resident or fellow directly after surgery (catheter use, intraoperative blood loss, bladder perforation and after discharge: length of stay, transfusion other complications	operating surgeon	more senior residents were involved in the complications, likely secondary to their disproportionate roles in more difficult resections. No statistical tests reported	case series	10.2

Palan 2009 [50]	7 hospitals, January 1999 - January 2002	Surgery	all levels of registrars performed 528 total hip replacements	1501 total hip replacements (1367 unilateral and 67 bilateral)	junior and senior registrars, of which senior registrars most likely have passed their fellowship examination	specialist consultants performed 973 total hip replacements	change in Oxford hip score (validated 12item instrument to assess function and pain) postoperative and after 5 years, dislocation, revision surgery, operating time, length of stay	trainer versus trainee (junior or senior registrar) Trainee outcomes were also compared according to training years	Higher oxford hip scores were observed after 1yr, 4yrs and 5yrs for more senior registrars ($p<0.01$). No differences between registrars and consultants on change in oxford hip score ($p>0.37$)	prospective cohort study	13.5
Sagarin 2005 [51]	29 university affiliated emergency departments, September 1996 - June 2001	Emergency medicine	emergency medicine (EM) residents	5768 initial attempts of EM residents	endotracheal intubation attempts in airway management	other resident years or specialists of EM or other specialties.	successful intubation defined as an endotracheal tube placed through the vocal chords. No follow-up.	training year of EM residents	success of initial intubation attempts increased over the first 3 years of residency (postgraduate year 1: 40% (95% CI 5%-85%), year 2: 77% (95% CI 63%-87%), year 3: 81% (95% CI 75%-86%), year 4+: 82% (95% CI 71%-91%), attending physician: 75% (95% CI 70%-79%)	case series	11.4

AOR Adjusted Odds Ratio; CI Confidence Interval

Table S1d. The effect of level of experience of residents on patient outcomes: Residents compared to faculty

First author and publication year	Setting	Specialty	Participants	Sample size	Teaching interventions	Comparison	Patient outcomes	Other outcome measurements	Effect	Study design	MERSQI score
Acun 2004 [52]	One teaching hospital, April 2001 - May 2003	Surgery	2 postgraduate year 2 surgical residents performed 74 operations under supervision	152 patient undergoing near-total thyroidectomies	postgraduate year 2 surgical residents under the direct supervision of an attending surgeon	2 attending surgeons with the same technical experience in thyroid surgery performed 78 operations	temporary or permanent vocal cord paralysis rate, temporary or permanent hyperparathyroidism rate, hematoma, bleeding, wound infection, seroma, mortality	primary surgeon	residents in training under supervision show no difference in complication rates compared to those performed by experts (*p*>0.05)	prospective randomized study	15
Auerbach 2008 [53]	Academic and private practice during 2000 and 2006.	Surgery	one surgeon assisted by fellows and residents (year 4-5) in academic practice (Group 1)	303 idiopathic scoliosis operations in adults using four different procedures	teaching versus non-teaching cases	One surgeon assisted by junior/senior attendings in private practice (Group 2)	age, gender, diagnosis, hospital, surgical assistant level, type of procedure, estimated blood loss (EBL), number of transfusions, duration of surgery, immediate postoperative major curve correction, length of fusion early complications.	within group comparisons and differences between fellows and residents were compared, but data not shown	longer operative times were observed in Group 1 for the less commonly performed procedures (33-48 minutes, *p*<0.01) and in the least common procedure more EBL was found for Group 1 (1668 ± 1182 versus 1273 ± 675, *p*=0.0016)	retrospective care series	14.4
Barnes 2006 [54]	Oculoplastic Clinic, when the study was performed is not reported.	Ophthalmology	resident or fellow compared to attending supervising physicians	55 consecutive cases of involutional entropion surgery	residents performed supervised surgery after observing and assisting at least 4 lateral tarsal strip and sutures for involutional entropion	fellow or attending operated	recurrence of entropion (horizontal eyelid laxity measurements in millimeters), suture granuloma's, ectropion	surgeon grade and experience: presented in the tables as resident, fellow or attending as operator	the operation was equally effective when performed by residents (1 recurrence, 1 medial ectropion), fellows (0 recurrences, 2 granulomas) or attending surgeons (0 recurrences, no adverse events) (*p*>0.4)	prospective case series	13.8
Baron 2008 [55]	Tertiary teaching centre, December 1997 - April 2004	Surgery	residents (years 4-6) under supervision	241 esophagectomies	Trainees performing the majority of the case were recorded as the first surgeon	The same consultant always present (scrubbed) in theater	postoperative (morbidity, mortality, stay in intensive care, hospital stay); pathological details and grade of disease; 15 months follow-up (recurrence and survival)	first surgeon and assistant; patient demographics; preoperative (type of malignancy and whether chemotherapy was given); operative (first surgeon and assistant, type of procedure) data registered	more leaks from stapled anastomoses by residents (10/84, 19%) compared to consultant cases (5/157, 5%) (*p*<0.01), however this was not reflected in an increased mortality (*p*=0.23)	retrospective case series	13.8

Borowski 2007 [56]	17 hospital in the Northern Region Colorectal cancer Audit group, between 1998 and 2002	Surgery	656 surgeries by an unsupervised trainee, 1578 surgeries by a supervised trainee	7411 (90%) of 8219 patients who underwent colorectal cancer surgery were included	Audit of all operations	140 consultants performed 4889 colorectal surgeries	operative mortality, anastomotic leak and long term survival (5 years)	primary surgeon	supervised trainees compared to consultants had a similar mortality (OR 0.90, 95% CI 0.71-1.16, *p*=0.418) and survival (HR 0.96, 95% CI 0.89-1.05, *p*=0.378)	cohort study	15
Chaudhuri 2006 [57]	1 Oct 2001 - 30 June 2003 in one cardiothoracic centre	Thoracic surgery	trainees yrs 1-3 and trainees yrs 4-6	115 trainee led lobectomy surgeries	surgical training program of 6 years	213 consultant led lobectomy surgeries	lobe removed, in hospital mortality, bleeding, respiratory complications, pneumonia, air leak from thoracostomy, lobar collapse, empyema, bronchopleural fistula, postoperative inotropic support, pulmonary embolism, myocardial infarction, renal function, cerebrovascular accident, transient ischemic attack	trainee as first operator and training year of trainees	if well supervised, residents' mortality rate (3.5%) was similar compared to consultants (2.8%) (*p*=0.83), survival rates at one year were not significantly different between trainees (82.6%) and consultants (81.7%) (*p*=0.83).	case series	13.8
Chiu 2006 [58]	One hospital, January 1998 - December 2003	Surgery	2 senior residents with experience in at least 50 open appendectomies	506 appendectomies with attendings' supervision	residents were first trained and later evaluated as they performed laparoscopic appendectomy under supervision	5 attendings with at least 7yrs experience performed 558 appendectomies in which residents assisted	operative time, conversion to open appendectomy, resumption of oral intake, hospital stay, frequency of analgesic injection, complications (wound infection, ileus, abscess) mortality	first surgeon and assistant	no differences between supervised residents and attendings including total complications (4.66% versus 3.64%, *p*>0.05) or mortality (0% versus 0.2%, *p*>0.05)	case series	12.6
Drain 2007 [59]	One hospital, 2000 - 2002	Surgery	junior specialist registrars (SpRs) years 1-3 and senior SpRs years 4-6 performing cardiac surgery under direct supervision	2079 cardiac operations	trainees start by observing and assisting and rapidly (within 3 months) progress to performing parts of the procedure.	junior SpRs, senior SpRs and consultants were compared	Euroscore mortality (all patients were prospectively risk stratified), total blood loss, blood loss after 12 hours and 'take-back' to the operating room for investigation of bleeding	operating surgeon status	no difference in blood loss (total and after 12hours) adjusted for Euroscore (*p*=0.48 and *p*=0.33), but take-back rate differed: junior trainees 9%, senior trainees 10.8%, consultants 6.8% (*p*=0.03)	retrospective case series	13.8
Ekanem 2008 [60]	University hospital, January 2000 - December 2001	Obstetrics and Gynecology	medical officers with at least 3 years of medical practice of which one year in Obstetrics and Gynecology	349 cases of emergency caesarean sections	training program in obstetrics and gynecology	senior registrar or consultant	demographics, antenatal booking status, pre and post operative packed cell volume < 30% was regarded as anaemic, intra operative blood loss, morbidity and mortality	participation of registrar, senior registrar and consultant in both obstetrics, gynaecology and anesthesiology to assess the medical team	mortality was only observed in residents' cases. Morbidity rates and length of stay were significantly higher for unsupervised (more junior) residents (*p*<0.007)	retrospective case series	11.4

Ferguson 2006 [61]	Not reported	Thoracic surgery	four trainees with at least 50 anatomical pulmonary resections via thoracotomy	276 video assisted thoracoscopic (VATS) lobectomy cases	VATS lobectomy training program	one self-trained consultant	lobe removed, operating surgeon, operative time, blood loss, preoperative lung function, complications, morbidity, mortality, pathology, postoperative stay	230 consultant cases were divided into 5 groups of 46 patients and the trainees also operated on 46 cases, so 6 even group sizes	VATS lobectomy can safely be done by trainees under supervision, with only a significantly longer operation time (159 minutes versus 137 minutes, *p*=0.0005)	retrospective cohort study	13.8
Filippi 2010 [90]	One level-one trauma center, 1 January 2006 - 31 December 2007	Radiology	radiology residents	317 magnetic resonance angiogram (MRA) studies	before taking call, residents must complete 1 month neuroradiology with experience interpreting MRA (with weekly teaching conferences) and didactic lectures at the start of the year on emergent neuroradiology studies	attending neuroradiologists	classified as false positive (normal as abnormal); false negative (failure to diagnose an abnormality); true-positive (infarct as an infarct); true-negative (normal as normal). Readmission, intensive care unit admission, new surgery, initiation of med treatment, new examinations were used as a change in clinical management	residency years 1-4 (n=22)	higher discrepancy rates than expected (10.4%), however non-significant (*p*=0.23) and without negative effects to patient care because of rapid turnaround time	case series	10.8
Fischer 2006 [62]	Tertiary teaching institution, January 2001 - October 2004	Surgery	138 operations performed by supervised residents	164 pancreatico-duodenectomies	general surgery training program	26 operations were performed by the attending	perioperative mortality (within 30 days), reoperation, pancreatic fistula, intra-abdominal haemorrage requiring a blood transfusion, intra-abdominal fluid collection, myocardial infarction, pneumonia, gastrointestinal bleeding, intensive care stay > 7days, hospital stay	resident participation (in the analysis the resident was not designated the 'primary surgeon' when the attending took over)	instead of group comparisons, outcomes were compared to the literature: mortality slightly higher (2.2% versus 1.9%), operation time longer (489 versus 431 minutes), blood loss slightly higher (1274ml versus 1193ml), fistula lower (6.5% versus 9.9%), length of stay shorter (13.5 versus 17.1 days)	prospective case series	11.4
Gueret 2007 [94]	University hospital, when the study was performed is not reported	Anesthesiology	supervised residents with < 10 fibre-optic intubations	46 fibre-optic intubations	residents performed the intubation under supervision (n=26), the senior anaesthesiologist took over if the number of attempts was higher than three, oxygen saturation decreased below 85% or intubation time exceeded 900 seconds	senior anaesthesiologist > 100 fibre-optic intubations performed 20 fibre-optic intubations, because a resident was not present in the operating room	SpO2 <90%, upper airway obstruction, laryngospasm, apnoea, fibre-optic intubation failure, tracheotomy, bleeding, hypertension, hypotension, tachycardia	resident or senior anaesthesiologist intubating	incidence of intubation failure was higher in the residents group (4/26 versus 1/20 for senior anesthesiologists, *p*=not significant), supervision is mandatory. Failure rate is comparable with literature reported outcomes	prospective case series	10.2

Gundevia 2008 [63]	Birmingham hospital, 1 Feb 2001 - 31 Jan 2004	Surgery	2 trainees in first year of higher surgical training	168 brachiocephalic, radiocephalic or brachio-basilic fistulas	comparison between patient outcomes in: (in)direct supervised trainees and consultants	4 consultants	fistula patency, 'used for dialysis' (defined by at least 6 consecutive sessions), primary failure, reason for failure, failure to mature, interventions required to salvage access when blocked, thrombosis, primary (assisted) patency	operating surgeon as defined in the theatre log books: trainee under direct supervision (scrubbed), independent trainees (in-hospital supervision), consultants	Survival analysis showed that grade of the operating surgeon did not relate to survival of the fistula’s (logranktest chi^2=3.1, *p*=0.38)	retrospective case series	13.8
Harris 2007 [64]	One hospital, 1 Jan 1998 - 31 Dec 2002	Orthopedic surgery	unsupervised trainees of varying experience	6361 orthopedic trauma operations	chi-square test to compare the two groups: supervised and unsupervised on classified complications	supervised trainees or consultants, unclassified who was first surgeon of the supervised group	complications classification: treatment error, expected risk, diagnostic error, where they have specified only 'treatment error' (fixation failure and mal deduction of fractures)	supervised or unsupervised	more complications in the supervised / consultant group compared to the unsupervised group (5.3% versus 3.3%, *p*=0.0001); Not corrected for case complexity	case series	11.4
Hollander 2006 [65]	General hospital, 1983 - 2002	Ophthalmology	residents	2718 cataract extractions	performing phacoemulsi-fication or standard extracapsular cataract surgeries	experienced surgeons	culture positive bacterial endophthalmitis, vitreous loss	topical preoperative antibiotics, resident or experienced surgeon	three cases of endophthalmitis, all who did not receive antibiotics. Vitreous loss was higher (6.7%, 95% CI 5.8%-7.7%) compared to reported rates in the literature of experienced cataract surgeons	retrospective case series	10.2
Hsu 2005 [66]	One academic medical centre, January 1998 - Augustus 2003	Surgery	4 surgeons performing laparoscopic Roux-en-Y gastric bypass (LRYGBP) with a resident assisting in the operation	281 LRYGBP cases	influence of residents assisting during the operation	attending or fellow operating	patient characteristics, concomitant operations, operative time, EBL, conversion, retro versus ante colic Roux, intra-operative complications, length of stay, intensive care admission, major complications also after discharge, whether a postoperative dilatation was necessary	training backgrounds of operating surgeons	residents compared to a fellow or attending as first assistant resulted in longer operative time (191,169, 177 minutes; *p*=0.014), higher re-admission rates (18.35, 2.56, 11.9;*p*=0.023) and intraoperative complication rates (10.1, 5.1, 4.8;*p*=0.048)	retrospective case series	13.8
Jain 2005 [67]	Academic medical center, July 1997 - July 2004	Surgery	1 HST (higher surgical trainee)	269 ambulatory laparoscopic cholecystectomy	62% of the operations were performed by a supervised HST	3 general surgery consultants	patient characteristics, morbidity, operating time, recovery nausea score, recovery pain score, satisfied patients, discharge, temporary transfer to the main hospital, conversion, bile duct injury, readmission	grade of the operating surgeon	HST needed longer operating times compared to consultants (47 versus 41 minutes, *p*=0.001), but there were no differences in other outcomes (*p*>0.5)	retrospective case series	10.2

Jaturapatporn 2007 [92]	One hospital, two weeks in December 2005	Family medicine	residents	1820 general practice assessment questionnaires (GPAQ)	3 year training program in family medicine	faculty qualified in family medicine and general doctors: doctors without training in family medicine	GPAQ is a standardized satisfaction questionnaire which assesses doctor's communication skills, patients' knowledge of self-care after consultation and overall satisfaction	frequency of visits, helpfulness of receptionist, access to healthcare, continuity of care, level of experience of doctor	Overall patient satisfaction was not statistically different between the three groups of practitioners (79.73-80.86 points, *p*=0.469)	case series	9.5
Jeganathan 2009 [68]	Tertiary referral centre, June 1994 - June 2006	Thoracic surgery	17 trainees performed 45 operations under consultants' supervision	252 thoracic oesophageal resections	number of trainees participating in the operations and their training background was not reported	5 consultants performed 207 operations	major complications (tracheostomy, re-intubation, commencement of inotropes, low cardiac output, renal impairment) surgical complications (wound dehiscence, anastamotic leak), mortality and 1- and 5-year survival follow-up	consultant or trainee operating	There was no statistically significant difference in in-hospital mortality between consultants (4.3%) and trainees (4.4%) (95% CI 0.84-1.26, *p*=0.61).	cohort study	12.6
Kanakala 2009 [69]	Northumbrian Upper Gastro Intestinal Team of Surgeons (NUGITS), 2002	Surgery	trainees under supervision of a consultant	51 patients underwent laparoscopic cholecystectomy (LC) 62 patients underwent laparoscopic inguinal hernia repair (LIH) by trainees	laparoscopic training courses for trainees early in their training with simulator-based training, hands-on experience with detailed constructive feedback after the operation	51 patients underwent LC and 62 patients LIH by consultants	per-operative events, post-operative complications, operative time, length of hospital stay, readmission	trainee or consultant operating	trainees required more time compared to consultants (LC: 72.63 versus 39.66 minutes, *p*<0.001, LIH: 64.55 versus 44.56 minutes, *p*<0.001) but other (post-operative) patient outcomes were not statistically significant between both groups (*p*>0.05)	prospective case-control study	13.8
Karagounis 2006 [70]	One institution, July 2002 - July 2004	Thoracic surgery	2 trainees with experience of 100 on-pump coronary artery bypass graft (CABG), performed 125 off-pump CABG as primary surgeon	323 off-pump CABG	selected patients were used to ensure gradual training possibility	One consultant performed 198 off-pump CABG cases	intra- and postoperative data (total complications, re-sternotomy for bleeding, mediastinal infection, postoperative laparotomy, tracheostomy, stroke, haemofiltration, intensive care stay, hospital stay, 30-day death	primary surgeon	Patient characteristics differed between consultant and trainee cases. Complex off-pump CABG procedures can safely be performed by trainees in a teaching setting	retrospective case series	13.8
Kauvar 2006 [71]	One center, 1 July 2002 - 30 June 2004	Surgery	Year 1-3 'junior residents' and year 4-5 'senior residents' under supervision	315 laparoscopic cholecystectomy cases	impact of attendings' experience on operative time of his/her junior residents (hypothesis: no impact)	Attendings were divided by trained before (n=7; mean post graduate years of 21) or after (n=8 mean post graduate years of 4) introduction of laparoscopic surgery	time from incision to skin closure, diagnosis, conversion, complications (duct injuries and leaks, bleeding requiring transfusion, intra-abdominal infectious complications or injury to intra-abdominal structures)	resident year, staff seniority. Inflammation was reported because it influences operative time	junior residents had longer operative times (86-88 versus 67-73 minutes, *p*<0.05) and higher complication rates (6.3% versus 0.79%, *p*=0.04) compared to senior residents, regardless of the seniority of the supervisors	retrospective case series	12.6

Khan 2008 [72]	Low-volume rural district general hospital, August 2003 - February 2006	Surgery	1 specialist registrar and 1 senior house officer (trainees) performed 21 operations under supervision	102 elective colorectal cancer resections	impact of surgical training on outcomes following elective colorectal resections for cancer	3 consultants, 1 staff grade surgeon	22 pre- and postoperative outcomes: type of resection, operative time, intensive care unit admission, mortality, repeat laparotomy, anastamotic leak, length of stay, respiratory complications, cardiovascular complications, wound infections	trainee/staff grade surgeon/consultant as primary surgeon and the level of the supervising specialist	survival was significantly poorer in consultant cases compared to trainee cases (hazard ratio 1.17, standard error 0.49, *p*=0.02), most likely due to case mix	retrospective case series	12.6
Lim 2006 [73]	Day-case unit, March 1996 - October 2003	Surgery	15 higher surgical trainees (HST) under supervision of a consultant	840 (572 unilateral and 134 bilateral) transabdominal preperitoneal herniorraphies	evaluation of a day-case laparoscopic herniorrhaphy program in a purpose-built day-case unit separate from the main hospital site	3 consultants	number of procedures, persistent symptoms, patient satisfaction, return to normal activity. Assessed on discharge and after two weeks	supervised HST were compared to consultants and HST were divided into 3 groups according to the number of operations they performed	operating time of HST significantly decreased after >40 operations compared to consultant cases (41.39 + 1.17 versus 41.4 + 0.87, *p*=0.31) no other differences in patient outcomes	case series	13.6
Marinis 2010 [74]	One university hospital, January 2005 - December 2007	Surgery	postgraduate year 5 and 6 residents under supervision	90 outpatient laparoscopic cholecystectomy (OLC) operations were performed by residents	outpatient day surgery unit in which higher surgical trainees work under supervision of a consultant	consultants performed 20 OLC operations	patient discharge on the day of surgery, complications, readmissions, morbidity, mortality, patient overall satisfaction and staff manners, on a visual analog scale	supervised higher surgical trainees (HST) were compared to consultants	OLC is clinically effective and can be performed safely in a teaching hospital by supervised HST, but HST required more operating time compared to consultants (45.76 ± 7.29 versus 35.5 ± 6.38, *p*<0.001)	prospective case series	11.4
Messina 2010 [75]	January 1998 - January 2006; follow-up was carried out December 2008 - March 2009	Thoracic surgery	4 supervised trainees	1333 off-pump CABG surgeries (977 by expert surgeons and 356 by trainees)	Off-pump coronary artery bypass graft (CABG) training	3 expert surgeons	patient characteristics, recurrent angina, myocardial infarction, EUROscore, cardiac catheterization, percutane transluminal coronary angioplasty or re-CABG, survival, ejection fraction	supervised trainee or surgeon as first operator	trainees had similar results as compared to expert surgeons, 1-2 and 4 year event-free survival: 98.5% versus 99.7%, 97.6% versus 98.8 and 97.4 versus 94.3% (*p*=0.4)	cohort study	13.8
Meyer 2009 [91]	1 January 2006 - 31 December 2007	Radiology	radiology residents	538 CT angiographies ordered during after hours	preliminary interpretations of radiology reports and the final reports of neuroradiology attending physicians	attending neuroradiologist	false-negative (failure to recognize abnormalities) or false positive (misinterpreting normal as abnormal)	major discrepancies were considered to potentially adversely affect outcome, change management or incur disability or death	discrepancy rates were higher than expected (13.6%) and especially among most junior residents (*p*=0.05), no adverse clinical outcome was detected	retrospective case series	10.8

Nguyen 2008 [76]	One hospital, June 2006 - September 2007	Urology	8 second year, 3 third year, 4 fifth year residents under supervision of the staff surgeon	267 elective office based vasectomies	patients were assigned to residents or staff primarily on whether the resident was at the clinic at the time	1 staff surgeon	visual analog scale (VAS) to assess pain immediately after vasectomy	residency year	VAS scores were 21.8 for residents and 19.5 for staff (*p*=0.25)	case series	13.8
Ogiso 2010 [77]	One teaching hospital, February 2001 - March 2008	Surgery	residents without prior experience in open colorectal surgery	46 patients with rectal cancer (35 intraperitoneal and 11 extraperitoneal) were operated by residents	stepwise training program on laparoscopic low anterior resection (LLAR) with three basic principles: selecting of appropriate cases, expert surgeons assist residents	91 patients with rectal cancer (40 intraperitoneal and 51 extraperitoneal) were operated by expert surgeons	blood loss, conversion to open surgery, complications, time to bowel recovery, distal margin of tumor resection, number of lymph nodes harvested, operative time	primary surgeon	resident surgeons perform the extraperitoneal procedure with longer operative time compared to expert surgeons (357 (240-558) versus 301 (160-615, *p*=0.047), but no other short-term outcome differences	prospective case series	11.4
Pajaanen 2010 [78]	One teaching hospital, 1996-2009	Surgery	12 third or fourth year residents performed 140 operations	317 adult patients were operated for inguinal hernia and 247 were follow-up for ten years	36 outpatient tension-free Lichtenstein hernia operations were performed by residents under supervision	141 patients were operated by the consultant	operation time, wound infection, wound hematoma, patient questionnaire scored on a visual analog scale (VAS): pain at rest and during physical exercise, testicular pain, need of pain-relieving medication, limitations in work or leisure-time activities, feeling of foreign body in groin, overall satisfaction at 1 month, 3 and 10 years post operation	primary surgeon	when selected cases are performed by residents after practicing under supervision, operative time is longer (62 ±18 versus 39 ±13 minutes, *p*<0.0001), but patient outcomes are similar (all not significant) compared to consultants	case series	11.4
Paul 2007 [93]	Five clinics with Planned Parenthood affiliation, 2003 - 2005	Family medicine	46 family medicine residents	1127 abortion procedures were done by residents under supervision	simulation training in manual vacuum aspiration, half day ultrasound practicum and 1 day per week placements at a Planned Parenthood clinic	experienced abortion providers of the Planned Parenthood clinics	complication rates (complications/number of surgical abortion procedures) and patient satisfaction	residents' satisfaction with the program	residents' complication rate (1.0%) is comparable to the rate of more experienced providers at Planned Parenthood (0.8%)	case series	10.2
Phillips 2007 [79]	One hospital, August 2003 - August 2005	Surgery	supervised trainees who performed >50% of the operation	83 patients with chronic sinonalsal symptoms	trainees had prior experience with this kind of surgery and were at a similar stage of training	consultant	SNOT-22 score (a tick-box questionnaire that assesses the severity of 22 key sinonasal symptoms) compared preoperative, at 3 month and 12 month postoperative	patient placement in 'trainee' or 'surgeon' group depended on trainee attendance in theater rather than any other factor	3- and 12-months post-operative SNOT-22 scores did not differ between trainees and consultants (*p*=0.92 and *p*=0.41 respectively)	case series	11.4

Pyle 2010 [80]	One university hospital, 2001 - 2007	Aesthetic surgery	chief residents work together with an attending surgeon readily available and frequently used	196 patients underwent 272 major cosmetic procedures	a chief resident serves as primary surgeon 1 day a week in a chief cosmetic clinic	outcomes were compared with literature reported outcomes	local complications (skin necrosis, wound dehiscence, wound infection, cellulites, scar asymmetry, hypertrophic scars, contour irregularities, hematoma, seroma and implant rupture), systemic complications (deep venous thrombosis, pulmonary embolism, myocardial infarction, sepsis, cerebrovascular accident, shock and death), revision rates	-	only descriptive data were reported: complication rates and revision rates, which compared favorably to other published reports,	retrospective case series	11.4
Robinson 2007 [81]	Private practice, 2002 and 2003	Orthopedic surgery	8 consecutive supervised trainees performed 88 total hip arthroplasties (THA)	142 patients (135 unilateral and 7 bilateral)	trainees worked with the attending surgeon for 3 months in which they progressed through an orderly sequence of steps in THA	one attending surgeon	operative time, surgical assistance, transfusion requirements, length of hospital stay, technical variables (angles and alignment), cement filling defects, intraoperative complications, Harris Hip score at 6 weeks, 3 months and 1 year postoperatively	resident involvement in the operation and use of health care resources	residents cases took more time (89 ± 12 versus 72 ± 15 minutes, *p*<0.00001) and required extra surgical assistance (92% versus 23%, *p*<0.0001) but residents' cases were similar to attendings' cases on all other outcome measures	case series	13.8
Robson 2004 [82]	Two teaching hospitals, January 1, 1994 - December 31, 2001	Surgery	junior trainees and senior trainees performed 815 and 1777 inguinal hernia repair operations, respectively	4406 patients underwent inguinal hernia repair of whom 90 required reoperation for recurrence	junior trainees operated unsupervised (31) supervised by a senior trainee (336) or a consultant (448) and senior trainees operated supervised by the consultant (266) or unsupervised (1201)	1814 patients were operated by a consultant	mean follow-up of 3 years, recurrence rates	primary surgeon and supervisor	primary inguinal hernia repairs can be performed by unsupervised, well trained senior surgical trainees (*p*=0.3-1.0) and by junior trainees supervised by either consultants or senior trainees (*p*=0.2-1.0), have recurrence rates similar to those of consultant	retrospective case series	13.2
Sivaslioglu 2007 [83]	Two teaching hospitals, June 2003 - June 2004	Obstetrics and Gynecology	senior residents performed 28 transobturator tape (TOT) surgeries with the senior surgeon assisting and teaching	55 women with stress urinary incontinence underwent TOT	urinary incontinence surgery training	senior surgeon performed 27 TOT surgeries	operative time, intraoperative and postoperative complications, intraoperative blood loss, effectiveness of the TOT procedure	primary surgeon	there is no difference in complications or effectiveness of the procedure (*p*>0.05), but operative time is longer for residents compared to senior surgeons (27 (15-48) versus 13 (7-22) minutes, *p*<0.05)	prospective open label randomized study	15.6

Skrekas 2006 [84]	Academic medical center, April 2003 - June 2004	Urology	one urology trainee with limited laparoscopic experience	44 laparoscopic radical prostatectomy's	mentor-initiated training program	skilled laparoscopist with experience of > 200 cases	intraoperative data: operating time, estimated blood loss, complications; postoperative data: decrease in hemoglobin on day 1, pathologic stage, positive-margin rate, catheterization time, hospital stay, complications 30 days after surgery	trainee as assistant (group 1: 16 LRPs) trainee under supervision (group 2: 12 LRPs) trainee without supervision (group 3: 16 LRPs)	trainees under supervision needed longer operative time (*p*<0.001) compared to the mentor. Longer operative time and catheterization days (both *p*<0.001) when the trainee operated without supervision compared to the mentor	case series	12.6
Stoica 2008 [85]	One teaching hospital, January 1, 1998 - December 31, 2005	Thoracic surgery	6 senior-level cardiac surgery trainees in postgraduate years 3 to 6 performed 1054 cases under supervision	6931 patients underwent coronary bypass graft surgery, aortic valve replacement or a combination	medical students can match directly to the 6-year full-fledged stand alone cardiac training program	7 staff surgeons performed 5877 nonteaching cases	death, stroke, reoperation for bleeding, intra-aortic balloon pump inserted during the operation or postoperatively, new postoperative renal failure, deep sternal wound infection, readmission for cardiovascular ischemic events, prosthetic valve-related complications, dysrhythmias, congestive heart failure	primary surgeon	resident as operator was not associated with in-hospital mortality (AOR 1.09, 95% CI 0.75-1.58, *p*=0.66), composite outcomes (AOR 1.01, 95% CI 0.53-1.34, *p*=0.46) or late death or hospital readmission (AHR 1.05, 95% CI .094-1.17, *p*=0.42)	case series	13.8
Teoh 2007 [86]	University hospital, July 1997 - December 2004	Surgery	3rd - 5th year surgical trainees operating in the presence of a trained specialist surgeon performing 46 operations	146 acute laparoscopic cholecystectomies for acute cholecystitis	Trainees had to attend a 3-day basic laparoscopic surgery course and be proficient in elective cases before being allowed to perform emergency laparoscopic cholecystectomy	43 specialist general surgeons performing 100 operations	in-hospital mortality (<30d), length of hospital stay (from admission as well as from operation), bile duct injury, bile leakage, retained biliary stones, conversion to an open cholecystectomy, duration of the operation, complication rate	trainee or specialist general surgeon	there were no differences between trainees and specialists on duration of operation (*p*=0.24), hospital stay (*p*=0.79), number of conversions (*p*=0.61) or complication rate (*p*=0.15)	case series	12.6
Wan 2007 [87]	University hospital, January 2002 - October 2006	Thoracic surgery	3 supervised trainees with two years of thoracic surgical training performed 60 operations	111 patients scheduled for video-assisted thoracic surgery (VATS) major lung resection	Trainees performed all procedures being supervised throughout the procedure	experienced self-trained VATS thoracic surgeons performed 51 operations	postoperative outcomes, hospital stay, blood loss, conversion, duration of chest drainage, thirty-day mortality rates, complications (pneumonia, air leak, empyema, cardiac disorders and renal failure)	trainee or consultant led VATS	trainees required more operation time compared to consultants (162 (112-212) versus 136 (87-184) minutes, mean difference -26.9, 95% CI -47.1 to -6.6, *p*=0.01*)*, but all other outcomes were similar (*p*>0.4)	case series	13.8

Wong 2007 [88]	Two university-affiliated teaching hospitals, 15 months study period	Surgery	5 specialist surgical trainees and one basic surgical trainee performed 92 laparoscopic appendicectomies (LA) and 122 open appendicectomies (OA)	143 LA and 222 OA	the consultant was on-call (not physically present in the operating room) available to attend in person if required	9 consultants performed 45 LA and 85 OA	operative time, conversion to open appendicectomy, complications, length of stay, readmission and mortality	pathology found at operation, type of appendectomy, additional supervision (excluded for analysis), grade of operating surgeon	unsupervised trainees had significantly longer operative times for both LA and OA compared to consultant surgeons ($p<0.05$), other patient outcomes did not differ ($p>0.2$)	case series	14.4
Woolson 2007 [89]	One teaching hospital, February 1996 - October 1999	Orthopedic surgery	performed 119 total hip arthroplasties (THA) and 72 total knee arthroplasties (TKA)	347 patients underwent 401 primary THA or TKA	a resident was first assistant in 66% of teaching cases and performed between one-third and one-half of the operation	one clinical professor, also a private practice surgeon, performed in private practice 111 THA and 99 TKA	operative blood loss, length of stay, operative time, outcomes on movement scores, early or late complications included dislocation, sublocation, reoperation, late revisions	follow-up time (average 59 month) and type of case (teaching versus private practice)	similar outcomes and complications for both groups ($p>0.18$), but operative time was longer for residents' participation compared to attending operating alone (72.5-79.8 minutes versus 61.0-73.2 minutes, $p<0.01$)	case series	13.8

AOR Adjusted Odds Ratio; OR Odds Ratio; CI Confidence Interval; HR Hazard Ratio; AHR Adjusted Hazard Ratio

Table S2. The effect of specific training interventions on patient outcomes

First author and publication year	Setting	Specialty	Participants	Sample size	Teaching interventions	Comparison	Patient outcomes	Other outcome measurements	Effect	Study design	MERSQI score
Barsuk 2009 [95]	Two adult ICU's in a tertiary hospital, August 2005 - March 2008	Intensive care	2nd and 3rd years internal and emergency medicine residents	98% of all central venous catheter insertions were done by residents	Pre-test on a simulator and use of a skills checklist and 2x2hours of education	post-test, until the test was passed	rates of catheter related bloodstream infection (CRBSI) per 1000 catheter days per month were measured, all positive blood cultures and medical records were identified and reviewed	3 groups: traditionally trained residents versus simulator trained residents in a MICU (medical ICU) versus a surgical ICU	a reduction of iatrogenic CRBSI was found when the simulator trained residents started working (regression ratio 0.16, 95% CI 0.05-0.44, p=0.001)	prospective cohort study	13.2
Buckley 2010 [96]	December 2006 - March 2008	Intensive care	residency and fellowship curricula in a medical intensive care unit	one educational plan-do-study-act (PDSA) project of 5 PDSA projects	Quality improvement process to enhance the training program's educational effectiveness and clinical outcomes	evaluation of PDSA projects	severity adjusted mortality, sepsis specific mortality, iatrogenic pneumothorax, ventilator-associated pneumonia, ICU length of stay, catheter related bloodstream infections	individual role of trainees in clinical outcomes	iatrogenic pneumothorax rates decreased from 0.31% to 0.17% (p<0.001) and sepsis specific mortality rates decreased from 17.8% to 13.8% (p<0.001) after initiation of the PDSA projects	case series	10.2
Edelson 2008 [97]	Tertiary care facility, March 2006 - February 2007	Internal medicine	team led by the on call cardiology resident with 1 or 2 interns and 0 or 2 medical students and other critical care personnel	123 patients were resuscitated	Resuscitation with Actual Performance Integrated Debriefing (RAPID) with weekly debriefing sessions	historical cohort in which a similar feedback-delivering defibrillator was used	return of spontaneous circulation, survival to discharge, shockable, time and location of arrest, patient demographics	trainee level (individual trainee performance could not be assessed)	resuscitation metrics improved (p<0.03) and the percentage of return of spontaneous circulation improved from 45% to 59% (p=0.03), but survival to discharge was indistinguishable (7% to 9%, p=0.69)	prospective case-control study	13.8
Ganzer 2008 [98]	Not reported	Urology	3 trainees from 2 training centres	1833 endoscopic extraperitoneal radical prostatectomy's (EERPE) (first 245 and 288 at the training centres and 1300 at the teaching centre)	Residents studied multimedia material and practice in dry and wet laboratory courses. Assisting in at least 5 EERPE procedures and thereafter performing supervised EERPE at their training centre	large series from the main teaching centre	Clavien classifications for operative complications and a self-administered patient questionnaire on incontinence 3- and 6-months following the surgery	-	Clavien classifications for operative complications were similar (p>0.22) and 6-months post-operative continence was 74.3% and 75% of patients in both groups	case series	13.2

Isaacson 2008 [99]	Tertiary children's hospital, 1997 - 2007	ENT	one surgeon supervising residents	> 10.000 tube insertions	A defined surgical protocol for tympanostomy tube insertion for residents under supervision	reduce major complications to the Six Sigma level (3.4/1.000.000)	sensorineural hearing loss (SNHL), injury to major vascular structures, disruption of the ossicular chain, loss of the tube in the middle ear, tympanic membrane tears, irreversible tube occlusion	major complications: canal laceration with bleeding, injury to the tympanic annulus, partial loss of the tube in the middle ear; minor complications: poor head positioning, inadequate cerumen removal, inadequate myringotomy, easily controlled bleeding	major and minor complications were within the Five Sigma level, but impact of the defined protocol could not be proven due to lack of comparison	case series	11.4
Lien 2007 [100]	General hospital, December 1990 - July 2004	Surgery	residents	5200 laparoscopic cholecystectomy's	Accident analysis --> Strategy development --> Database evaluation	attending doctors	operation time, indwelling drainage tube or not, conversion rates before and after introducing new strategies	attending or resident as surgeon	residents took more time compared to attendings (79.8 $\pm$ 30.9 versus 103.4 $\pm$ 46.3 minutes, p=0.003), but no difference in indwelling drainage tubes occurred for acute and elective procedures (p=0.269 and p=0.577) after the strategy development	cohort study	13.8
Lienard 2010 [101]	Not reported	Different specialties	88 residents from different specialties, five had attended a communication course in the last year	750 inpatient visited during a half-day clinical round at baseline (n=390) and after 8 months (n=367)	40-hour communication skills training program with role play and direct feedback spread bimonthly over 8 months	residents on a waiting list	patient satisfaction measured on a visual analogue scale (VAS) - from 'poorly satisfied' to 'extremely satisfied'	utterances were analysed from actual inpatient visits' audiotapes	patient satisfaction was not differ at baseline (p=0.366), but patient satisfaction was higher for the trained group compared to the waiting list residents (p=0.047)	randomized controlled trial	15
McMahon 2010 [102]	One teaching hospital, during one year	Internal medicine	Two teams of the new integrated teaching unit (ITU) 2 residents, 3 interns and 2 attendings per team	3988 medical inpatients (1892 to the ITU teams and 2096 to the general medical service (GMS) teams	experimental inpatient-medicine service (patients were alternately assigned to an ITU of GMS team) in which residents under increased supervision were encouraged to take leadership	Two GMS teams 1 resident, 2 interns and multiple supervisors per team	patient demographics, primary and secondary diagnosis, length of stay, in-hospital death, cause of death, readmission, patient satisfaction after discharge	observation of intern activity (direct pt care, indirect care, education, other tasks) satisfaction of team members	length of stay was shorter for ITU teams with 0.5 days (p=0.002), but due to possible bias the validity of this finding is unclear, no other differences in patient outcomes. The ITU team reported overall higher satisfaction (78% versus 55%, p<0.01)	case-control study	13.8

Pandey 2007 [103]	One institution, 1996 - 2005	Surgery	71% of operations by higher surgical trainees and 12% by basic surgical trainees	330 appendectomies and 78 hemicolectomy's	hemicolectomy approach which directly follows the appendectomy approach used to educate trainees under supervision	-	operation time, conversion, wound infection, urinary tract infection, readmission, length of stay, reoperation, perforation, anastomotic leak, obstruction, renal failure, mortality	-	no comparison was made, but trainees required more time for the laparoscopy	case series	10.2
Papadimos 2008 [104]	Trauma level 1 university hospital, 1 January 2003 - 31 December 2007	Intensive care	anesthesiology or surgical residents years 2-6	85 observed resident central venous catheter insertion (CVC)	intensivist supervision of the use of 7 requirements to ensure maximal sterile barriers (MSB) in CVC	historical control period (1 January 2001 - 31 December 2003)	catheter-related bloodstream infections (CRBSI)	observed breaks of MSB requirements	with additional supervision, there was an annual downward trend of CRBSI rates in 2004-2007 (Z=4.576, *p*<0.0001) as well as a monthly trend (*p*=0.028)	case-control study	12.6
Rogers 2009 [105]	Single residency-training site, 1998 - 2008	Ophthalmology	Group 1: 3rd yr residents performing 823 cases before a curriculum change	1832 phaco-emulsification cataract surgeries	curricular change included: wet lab and simulator training and backing into cases of senior residents during the first year, formative feedback and deliberate practice during 2nd year	Group 2: 3rd year residents performing 1009 cases after a curriculum enhancement	Sentinel event (a posterior capsule tear or vitreous loss)	data were adjusted for surgical experience	reduction in sentinel event complication rates before and after the enhanced curriculum (7.17% to 3.77%, *p*=0.008)	case-control study	13.8
Schmidt 2008 [106]	Academic tertiary care hospital, 1 November 2006 - 15 April 2008	Anesthesiology	residents performed 207 intubations without an attending anesthesiologist	322 emergency intubations	residents have a minimum of 6 months anesthesia experience and performed 162-344 intubations	supervised residents performed 115 intubations	mortality, ventilator-free days, patient returning to home/nursing home or rehabilitation facility and complications (esophageal intubation, traumatic intubation, aspiration, dental injury, endobroncheal intubation)	attending supervision was recorded by an always present respiratory therapist	overall complications were lover for supervised residents: 7 (6.1%) compared to no supervision(45 (21.7%), (*p*=0.0001)	case series	12.6
Thomas 2007 [107]	Residency continuity clinic, Mayo Clinics, 2003 - 2004	Internal medicine	39 residents received audit, feedback and patient reminders	483 diabetic patients	two 1-hour sessions introducing registries, quarterly residents were provided with registry-generated feedback on their performance.	39 residents received usual education consisting of faculty review of diabetes care among patients supervised with the resident	hemoglobin A1c, lipid monitoring: low density lipoprotein (LDL) cholesterol, blood pressure control	process outcomes (HbA1c monitoring within 6 months and LDL cholesterol monitoring within 1 year)	there were no intermediate clinical outcome differences between intervention and control group (*p*>0.1), but process outcomes improved (*p*<0.02)	randomized controlled trial (brief report)	15

Warm 2008 [108]	One academic health center, November 2005 - October 2007	Internal medicine	69 categorical based residents in a resident ambulatory practice	4983 patients were seen in the ambulatory practice	as part of the Education Improvement Program (EIP) a year-long continuous ambulatory group-practice experience was implemented called the long-block	patient satisfaction one year before the long-block	patient satisfaction measured using publicly reported Press-Ganey surveys and an adapted Consumer Assessment of Health Plans Survey (CAHPS), diabetes, hypertension and preventive care measures	residents' satisfaction with the program	patient satisfaction increased for the total resident practice score (78-79.3-80.7) as well as the provider specific score(80.7-83.3-84.5) and residents' satisfaction also increased (73.23 to 87.50, *p*=0.0016)	case series	11.4
Wayne 2008 [109]	One university affiliated hospital, January 2004 - June 2004	Internal medicine	38 second-year residents received four 2.5-hour simulation-based educational sessions	20 events were led by simulator trained residents and 28 by non-simulator trained residents	simulation-based sessions featured the deliberate practice of advanced cardiac life support (ACLS) scenarios using a human patient simulator	40 third-year residents were not trained on the simulator	survived event, mean post event survival to death or hospital discharge in hours, survived to hospital discharge	correct adherence to the American Heart Association (AHA) guidelines for ACLS	events led by simulator trained residents compared to non-simulator trained residents resulted in similar outcomes (*p*>0.11), but adherence to guidelines was better (68% versus 44%, *p*=0.001)	case-control study	13.8
Woo 2007 [110]	County psychiatric facility affiliated to a University hospital, 2003 - 2007	Psychiatry	no residents participated prior to the new program	915 inpatients (2003-2004) and 853 inpatients (2005-2006)	a new residency program (5 residents per year)	first year of full implementation (5 first year and 5 second year residents present)	length of stay, 24-hour transfers, 30-day readmissions, use of seclusion and restraints, patient survey question 'did the psychiatrist talk to you and help you with your problems'	utilisation of resources	length of stay increased with 1 day (*p*=0.02), 30-day readmission increased from 3.5% to 5.6% (*p*=0.04) and patient satisfaction dropped from 76.9% to 70.0% (*p*=0.01)	case-control study	13.8
Ziemer 2006 [111]	One primary care clinic, January 1, 2000 - December 31, 2002	Internal medicine	345 internal medicine residents	4038 African American patients with diabetes mellitus	residents in the intervention group received reminders providing patient specific recommendations, feedback on performance every two weeks or both	no intervention	HbA1C	action undertaken by the resident to optimize HbA1c control	feedback on performance given to medical resident primary care providers lowered HbA1c levels (regression coefficient -0.2357, *p*=0.003)	randomized controlled trial	15

CI Confidence Interval

Table S3. Post-resident patient outcomes according to where the resident trained

First author and publication year	Setting	Specialty	Participants	Sample size	Teaching interventions	Comparison	Patient outcomes	Other outcome measurements	Effect	Study design	MERSQI score
Asch 2009 [112]	All non-federal acute care hospitals in Florida and New York between 1992 and 2007	Obstetrics and Gynecology	4124 physicians from 107 US residency programs of which at least 10 physicians could be identified	4 906 169 deliveries	43% of accredited Obstetrics and Gynecology residency programs in the United States	Comparison of top and bottom quintile of training programs	Maternal complications divided in 9 codes: laceration, hemorrhage, other (including infection and thrombotic complications) for vaginal delivery. Hemorrhage, infection and other (thrombotic complications) for cesarean delivery and composites for each and for overall	Licensing examination scores	Adjusted complication rates differed between top quintile (10.3%) and bottom quintile (13.6%) of residency training programs, absolute difference 3.3% (95% CI 2.8%-3.8%)	retrospective cohort study	13.8

CI Confidence Interval
